# Prevalence and outcomes of HIV‐1 diagnostic challenges during universal birth testing – an urban South African observational cohort

**DOI:** 10.7448/IAS.20.7.21761

**Published:** 2017-08-29

**Authors:** Karl‐Günter Technau, Ahmad Haeri Mazanderani, Louise Kuhn, Lucia Hans, Renate Strehlau, Elaine J. Abrams, Martie Conradie, Ashraf Coovadia, Ndileka Mbete, Pamela M Murnane, Faeezah Patel, Stephanie Shiau, Caroline T. Tiemessen, Gayle G. Sherman

**Affiliations:** ^1^ Empilweni Services and Research Unit, Department of Paediatrics & Child Health, Rahima Moosa Mother and Child Hospital, Faculty of Health Sciences, University of the Witwatersrand, Johannesburg, South Africa; ^2^ Centre for HIV & STIs, National Institute for Communicable Diseases, Johannesburg, South Africa; ^3^ Department of Medical Virology, Faculty of Health Sciences, University of Pretoria, Pretoria, South Africa; ^4^ Gertrude H. Sergievsky Center, College of Physicians and Surgeons, and Department of Epidemiology, Mailman School of Public Health, Columbia University, New York, USA; ^5^ Department of Molecular Medicine and Haematology, Faculty of Health Sciences, University of the Witwatersrand, and National Health Laboratory Service, Johannesburg, South Africa; ^6^ ICAP, Mailman School of Public Health, and Department of Pediatrics, College of Physicians & Surgeons, Columbia University, New York, USA; ^7^ School of Pathology, Faculty of Health Sciences, University of the Witwatersrand, Johannesburg, South Africa

**Keywords:** HIV‐1 PCR, early infant diagnosis, birth testing, indeterminate

## Abstract

**Introduction**: HIV‐1 polymerase chain reaction (PCR) testing at birth aims to facilitate earlier initiation of antiretroviral therapy (ART) for HIV‐infected neonates. Data from two years of universal birth testing implementation in a high‐burden South African urban setting are presented to demonstrate the prevalence and outcomes of diagnostic challenges in this context.

**Methods**: HIV‐exposed neonates born at Rahima Moosa Mother and Child Hospital between 5 June 2014 and 31 August 2016 were routinely screened at birth for HIV‐1 on whole blood samples using the COBAS® AmpliPrep/COBAS® TaqMan (CAP/CTM) HIV‐1 Qualitative Test, version 2.0 (Roche Molecular Systems, Inc., Branchburg, NJ, USA). Virological results were interpreted according to standard operating procedures with the South African National Health Laboratory Service. All neonates with non‐negative results were actively followed‐up and categorized according to HIV infection status as positive, negative, uncertain and lost to follow‐up (LTFU).

**Results**: 104 (1.8%) of 5743 HIV‐exposed neonates received a non‐negative birth PCR result, for which laboratory data were available for 102 (98%) cases – 78 (76%) tested positive and 24 (24%) indeterminate. HIV infection status was confirmed positive in 83 (81%) infants, negative in 8 (8%), uncertain in 5 (5%) and LTFU in 6 (6%) cases. The positive predictive value (excluding cases of uncertain diagnosis and inadequate testing) following a non‐negative HIV‐1 PCR screening test at birth was 0.91 (83/91; 95% confidence interval: 0.85–0.96). Neonates testing positive at birth had significantly higher viral load (VL) results than those testing indeterminate at birth of 4.5 and 3.0 log copies/ml (*p* = 0.0007), respectively. Similarly, mothers of neonates with positive as compared to indeterminate birth test results had higher VLs of 4.5 and 2.7 log copies/ml (*p* = 0.0013), respectively. Half of neonates with an indeterminate birth test were shown to be HIV‐infected on subsequent confirmatory testing, with time to final diagnosis 30 days longer for these neonates (*p* < 0.0001).

**Conclusion**: Indeterminate HIV‐1 PCR results accounted for a quarter of non‐negative results at birth and were associated with a high risk of infection in comparison to the risk of *in utero* transmission. Indeterminate birth results with positive HIV PCR results on repeat testing were associated with later final diagnosis. The HIV‐1 status remains uncertain in a minority of cases because of repeatedly indeterminate results, highlighting the need for more sensitive and specific virological tests.

## Introduction

Routine HIV‐1 polymerase chain reaction (PCR) testing at birth for all HIV‐exposed neonates was introduced into the South African Consolidated Guidelines in June 2015 in order to enhance access to care and thereby reduce HIV‐related morbidity and mortality [1,2]. Although targeted birth testing amongst high‐risk HIV‐exposed neonates had been part of the national testing guidelines since 2013 [3], implementation of the new guidelines has seen the volume of testing amongst neonates less than seven days of age increase by more than 40 times, with approximately 20,000 birth tests performed each month [4]. Importantly, whereas the rapid scale‐up of birth testing has been associated with earlier diagnosis of intrauterine‐infected neonates, there has also been an increase in the number of diagnostic challenges arising during the neonatal period.

Early infant diagnosis (EID) and treatment of HIV‐1 is important due to the rapid progression of disease and early HIV‐related morbidity and mortality [5–7]. Accurate diagnosis is critical to ensure that infected infants start antiretroviral therapy (ART) early and that uninfected infants are not unnecessarily exposed to life‐long treatment. The recent case of the “Mississippi baby”, and other similar cases, further highlights the importance of very EID on account of the potential for functional cure and loss of detectability associated with very early ART initiation. These cases also highlight the possibility that diagnostic difficulties at birth may hold important information for this field [8–10].

Although PCR testing methods used for EID have reported sensitivities and specificities nearing 100% [11,12], important limitations exist. High coverage of maternal ART and early infant prophylaxis, as well as early initiation of treatment in infants prior to receiving confirmatory test results, are associated with high‐level exposure to ART at the time of testing. This in turn has been associated with suppression of viraemia in infected infants and loss of detectability and uncertain results when using diagnostic assays [13–17]. Although the association between ART and indeterminate HIV‐1 PCR results has been highlighted, and recommendations made regarding the management of such infants, further research is required to ensure timely definitive diagnosis and successful linkage to care [18,19]. Waning antibody levels and seroreversion following early ART initiation are additional phenomena that make later diagnostic confirmation difficult [20–22]. In contrast to the possibility of loss of detectability, there is also concern that on account of the dramatic reduction in mother‐to‐child transmission in South Africa there will necessarily be a drop in the positive predictive value of all infant diagnostic testing methodologies, thereby increasing the risk of treating uninfected infants [23–25].

Hence, research is urgently needed to inform evidence‐based management of infants with uncertain and indeterminate HIV‐1 results during early infancy. We describe the prevalence and outcomes of diagnostic challenges associated with HIV‐1 PCR testing at birth within a single health facility in a high‐burden setting over a two‐year period.

## Methods

Neonates born to HIV‐infected mothers were enrolled at Rahima Moosa Mother and Child Hospital (RMMCH), a tertiary institution situated in Johannesburg, South Africa, with approximately 1000 deliveries per month and an antenatal HIV prevalence of 23% [26]. The cohort of all infants born between 5 June 2014 and 31 August 2016 who had an HIV‐1 PCR test at birth were included in the analysis. During this period, all HIV‐exposed neonates were routinely screened at birth for HIV‐1 using ethylenediaminetetraacetic acid (EDTA) anti‐coagulated whole blood samples obtained by phlebotomy in the hours following delivery and sent to a diagnostic laboratory for testing. Those with a non‐negative result were actively traced and followed up either at RMMCH or referred to local facilities if unable to return. Initially all neonates with a non‐negative laboratory HIV‐1 PCR result were initiated on combination ART and samples taken simultaneously for confirmatory testing on follow‐up. This practice was based on findings (from the previous version of the current assay) that neonates with an HIV‐1 PCR result at birth, whether positive or indeterminate, were invariably found to have a confirmed HIV‐1‐positive infection status [27]. However, after it was found that some neonates who tested indeterminate at birth had negative confirmatory testing, this practice was stopped and only neonates with a clearly positive virological result were initiated on ART prior to awaiting confirmatory results. For those neonates following up at RMMCH, confirmatory testing was performed using the same qualitative HIV‐1 PCR assay and/or a viral load (VL) test on EDTA anti‐coagulated whole blood and plasma, respectively. Neonates with indeterminate or discordant results were retested at each clinic visit until a definitive HIV‐1 status was determined. Qualitative PCR testing outside of RMMCH was performed on either EDTA anti‐coagulated whole blood or whole blood dried blood spot (DBS) samples, depending on access to phlebotomy services. All mothers of neonates who underwent birth testing and received a negative result were recommended to follow up for additional testing to detect possible intra‐partum and post‐partum infection according to national guidelines [1].

## Laboratory methods

Qualitative HIV‐1 PCR and VL testing were performed at accredited (ISO 15189:2012) diagnostic laboratories using the qualitative and quantitative versions of the COBAS® AmpliPrep/COBAS® TaqMan (CAP/CTM) HIV‐1 Test, version 2.0 (Roche Molecular Systems, Inc., Branchburg, NJ, USA). The CAP/CTM is a total nucleic acid real‐time reverse transcriptase PCR assay that detects HIV‐1 proviral DNA and RNA on whole blood, and HIV‐1 RNA only on plasma, with a limit of detection of approximately 300 RNA copies/ml and limit of quantification of 20 RNA copies/ml, respectively [11,28]. All non‐negative virological results were interpreted according to standard criteria used within the National Health Laboratory Service (NHLS) to distinguish clearly positive from indeterminate results. All qualitative HIV‐1 PCR results with a cycle‐threshold value of ≤33 and a relative fluorescence intensity ≥5, and VL results with a quantified or higher than the linear range result (>7 log RNA copies/ml) were defined as clearly positive. Hence, all non‐negative virological results with a cycle‐threshold of >33 and/or relative fluorescence intensity of <5 and VL results where RNA was detected but below the linear range of the assay (i.e. <1.3 log RNA copies/ml or <2.0 log RNA copies/ml for those samples that required a 1:5 dilution due to inadequate volume) were interpreted as indeterminate [29,30]. As a means for controlling for sample swap, genetic profiling of short tandem repeat loci was performed using the PowerPlex® 16 HS System (Promega Corporation, Madison, WI, USA) on the birth and subsequent samples of a patient who had a clearly positive virological result at birth followed by negative results on subsequent clinic visits.

## Classifying HIV‐1 status

The final diagnostic status of infants who received a non‐negative HIV‐1 PCR result was classified as follows:

‐ Positive HIV‐1 infection status was based on two clearly positive virological results from samples taken at two different time points.
‐ Negative HIV‐1 infection status was defined as an isolated indeterminate result followed by at least two negative confirmatory virological results taken at two different time points whilst not on combination ART.
‐ Uncertain HIV‐1 infection status was defined as neonates with non‐negative virological results that did not meet criteria for confirmed positive or negative infection status after repeat testing.
‐ Lost to follow‐up (LTFU) was defined as patients who did not have sufficient follow‐up testing to meet any of the above criteria.

We examined whether maternal factors, including VL, CD4 cell count or duration of ART pre‐delivery, or infant factors, including age at screening test, age at diagnostic confirmation, VL and relation to commencement of daily dose nevirapine (ddNVP), were related to the screening or confirmatory testing outcomes in any way. All mothers identified as HIV‐infected who delivered at RMMCH between June 2014 and August 2016 were invited to sign a data sharing informed consent form, approved by the Human Research Ethics Committee of the University of the Witwatersrand (M130653, M140760, M140555 and M140639). Clinical and laboratory data, recorded on paper, were captured into a routine REDCap database [31]. Data were analysed using SAS (Version 9.4, SAS Institute Inc., Cary, NC, USA), and descriptive methods were used to present frequencies of events, medians and interquartile ranges (IQRs), the Cochran–Armitage Trend test to assess trends of indeterminate result outcomes and Kaplan–Meier method to assess time to diagnosis. We describe the events along the diagnostic process. Cases with complex or uncertain diagnostic events had file reviews and are presented as brief case reports.

## Results

A total of 5743 (91%) of the 6309 HIV‐exposed neonates born at the hospital were enrolled in the study of which 104 (1.8%) received a non‐negative HIV‐1 PCR result at birth. Of 102 (98%) neonates with laboratory data available, 78 (76%) were classified as positive and 24 (24%) were indeterminate according to laboratory criteria. After confirmatory testing, 83 (81%) infants were confirmed HIV‐1 infected, amounting to an intrauterine transmission rate of approximately 1.4%, and 8 (8%) infants were assigned a negative HIV‐1 infection status. The HIV‐1 status of an additional 5 (5%) infants remains uncertain and 6 (6%) were LTFU. The positive predictive value (excluding cases of uncertain diagnosis and inadequate testing) following a non‐negative HIV‐1 PCR screening test at birth (i.e. all detected virological results) was 0.91 (83/91; 95% confidence interval: 0.85–0.96), and this increased to 1.0 when using NHLS cutoff values to distinguish positive from indeterminate results.

Amongst the 83 infants who were confirmed HIV‐1 infected, 74 had a positive and 9 had an indeterminate HIV‐1 PCR result at birth (Table 1). Of the 74 neonates who screened positive, 72 had a retrievable confirmatory VL result, with a median value of 4.5 log copies/ml (IQR: 3.4–5.4), which was significantly higher than the 9 neonates with indeterminate results at birth who had a median VL of 3.0 log copies/ml (IQR: 2.8–3.2, *p* = 0.0007). All eight neonates who were diagnosed as uninfected had an isolated indeterminate screening result with at least two subsequent undetected virological results. Three of these infants repeatedly tested negative following all ART exposure cessation (including ddNVP prophylaxis and potential ingestion in breast milk of maternal ART) and five tested negative whilst still on ddNVP prophylaxis. Excluding the six infants who were LTFU, infection status could be clearly confirmed in the majority of cases (87 (91%) of 96 cases) by repeat virological testing on follow‐up. However, in 9 (9%) of 96 cases a clear diagnosis could not be made on immediate follow‐up on account of testing yielding repeatedly indeterminate virological results or negative results within the context of combination ART pressure. In four of the nine cases (cases a–d, Figure 1(a)), ongoing testing eventually confirmed a positive HIV‐1 infection status while in five cases (cases e–i, Figure 1(b)) the diagnosis remains uncertain despite further testing.

**Table 1:** Steps in establishing final HIV‐1 infection status of 102 infants with non‐negative birth PCR results

First PCR result		Second PCR result			Earliest VL result			Final HIV‐1 infection status,^†^ *N* (%)			
Birth HIV‐1 PCR test	*n*	Result	*n*	Age (days)^‡^	*n*	VL (log RNA copies/ml)^‡^	Age (days)^‡^	Positive	Uncertain	Negative	LTFU
Positive	78	Positive	68	2 (1–9)	66	4.48 (3.4–5.4)	2 (1–8)	68			
		Indeterminate	4	4, 4, 8, 40	4	<1.3^e^, 2.58, 4.09, 4.56	68, 8, 4, 4	3	1^e^		
		Not tested	6		3	4.30, 5.04, 6.62	1, 4, 94	3			3
		Total birth HIV‐1 PCR positive results				74 (95)	1 (1)		3 (4)		
Indeterminate	24	Positive	5	6 (6–12)	4	2.29, 2.96, 3.05, 4.45	12, 0, 6, 1	5			
		Indeterminate	7	8 (6–24)	6	TND^i^, 3.1 (3.0–3.2)	2 (1–8)	4^a^^–^^d^	2^g,i^		1
		Negative	11	7 (3–11)	10	TND (*n* = 9), 2.82^f^	8 (4–10)		2^f,h^	8	1
		Not tested	1		0						1
		Total birth HIV‐1 PCR indeterminate results				9 (38)	4 (17)	8 (33)	3 (13)		
Total, *n* = 102								83 (81)	5 (5)	8 (8)	6 (6)

†Positive HIV‐1 infection status was defined as two positive virological results from samples taken at two different time points. Negative HIV‐1 infection status was defined as an isolated indeterminate result followed by at least two negative virological results taken at two different time points whilst not on combination ART. Uncertain HIV infection status was defined as initial non‐negative virological results that did not meet either of the above criteria. Lost to follow‐up (LTFU) was defined as insufficient follow‐up testing to meet the above criteria. ‡Individual results are displayed for ≤4 cases and median (interquartile range (IQR)) for ≥5 cases. PCR: polymerase chain reaction; VL: viral load; TND: target not detected. The superscripts ^a^^–^^d^ in column Positive and ^e^^–^^i^ in column Uncertain indicate the cases presented in more detail in Figure 1(a,b), respectively.

**Figure 1:**
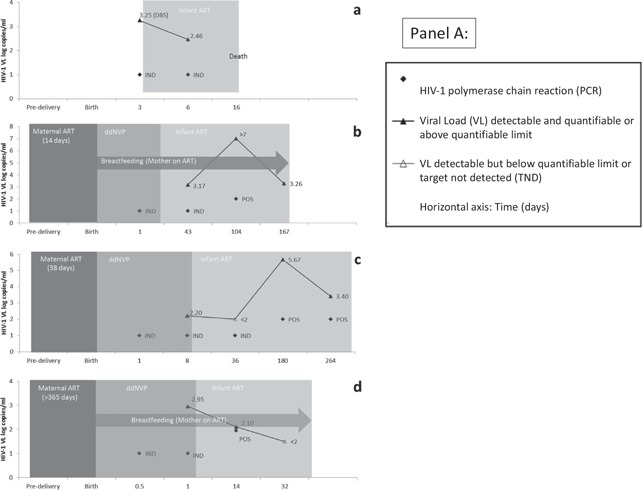
(A) and (B): HIV‐1 PCR and viral load (VL) results in cases with positive (a–d) and uncertain HIV infection status (e–i), respectively. The time periods for which maternal antiretroviral therapy (ART), infant prophylaxis of daily dose nevirapine (ddNVP) and infant ART were given are represented by progressively lighter shades of grey. HIV‐1 PCR tests were all done on whole blood and VL tests performed on plasma except where DBS is indicated. Due to space constraints some later repeat PCR negative or VL TND results were omitted (cases f–h). DBS: dried blood spot; POS: positive; IND: indeterminate; NEG: negative; ART: antiretroviral therapy.

**Figure 1:**
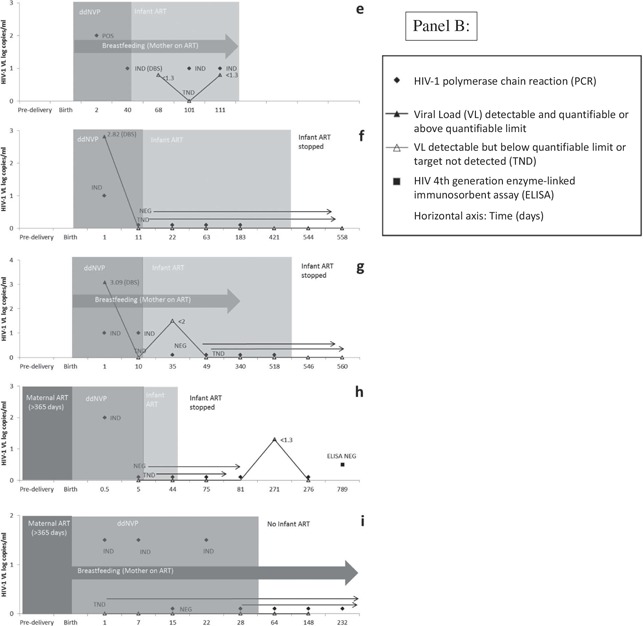
(continued).

**Figure 2:**
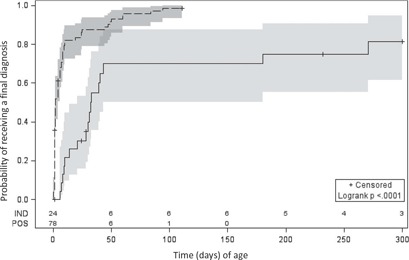
Kaplan–Meier curves of time to successful diagnosis (infected or uninfected) comparing infants with screening birth PCR positive (*n* = 78) to indeterminate (*n* = 24) results. POS: positive (dashed line), IND: indeterminate (solid line) HIV‐1 polymerase chain reaction (PCR) screening result with shaded areas representing 95% confidence intervals and numbers at risk above the *x*‐axis. Censoring occurred at last test where diagnosis remained uncertain or at last visit date where loss to follow‐up occurred.

Samples for HIV‐1 PCR screening at birth were taken at a median age of 14 h (IQR: 7–23) and were not significantly different (*p* = 0.52) between neonates with a positive birth result as compared to neonates with an indeterminate result (Table 2). However, time to diagnosis was significantly later for infants with an indeterminate screening result (*p* < 0.0001), which was confirmed as either infected or uninfected on a sample taken at a median age of 32 days (IQR: 14–180) by Kaplan–Meier analysis (Figure 2). For infected infants the time to confirmation was also significantly longer (*p* = 0.0004) for infants who had indeterminate HIV‐1 PCR tests at birth (31 days (IQR:14–43)) compared to infants with a positive birth test (2 days (IQR: 1–8)). Duration of maternal antenatal ART exposure was not significantly different between infants with a positive birth test as compared to infants with an indeterminate birth test, but maternal VL values were significantly different (*p* = 0.0013) with mothers of neonates with screening indeterminate results having a lower median baseline VL result (2.7 vs. 4.5 log copies/ml). There was a trend towards a higher CD4 count in mothers of neonates who tested indeterminate at birth but this was not statistically significant (*p* = 0.059). The probability of being confirmed as infected was 99% for infants with an initial screening positive result (one case remains with an uncertain diagnosis) versus 43% for infants with a screening indeterminate result (*p* < 0.0001). When stratifying neonates by final diagnostic status (positive, negative or uncertain), there was a significant difference only in maternal VL (*p* = 0.0008) when comparing those with a confirmed positive HIV‐1 status (*n* = 68) median 4.5 (IQR: 3.4–5.1), uncertain HIV‐1 status (*n* = 4) median 2.8 (IQR: 2.4–3.4) and confirmed negative HIV‐1 status (*n* = 7) median 1.9 copies/ml (target not detected‐3.9). Four (17%) of 24 neonates who tested indeterminate and 7 (9%) of 78 neonates who tested positive at birth (*p* = 0.28) died during the course of this study.

**Table 2:** Associations between screening birth HIV‐1 PCR results and maternal and infant factors

	HIV‐1 PCR positive	HIV‐1 PCR indeterminate	*p*
*N*	78	24	
No maternal ART exposure, *n* (column %)	25 (32)	5 (21)	0.29
Maternal ART exposure pre‐delivery, *n* (%)	53 (68)	19 (79)	
0–12 weeks	21 (40)	6 (32)	0.48
12–26 weeks	20 (38)	6 (32)	
>26 weeks	12 (23)	7 (37)	
Median (IQR) weeks ART exposure	16 (7–23)	18 (3–135)	0.61
Maternal viral load (VL) data available, *n* (%)^a^	61 (78)	19 (79)	0.92
Median (IQR) log copies/ml	4.5 (3.7–5.0)	2.7 (1.9–4.3)	0.0013
Maternal CD4 cell count data available, *n* (%)	75 (96)	22 (92)	0.37
Median (IQR) maternal CD4 cell count (cells/μl)^a^	280 (168–472)	406 (264–608)	0.059
Median (IQR) age (hours) when birth sample taken	13.7 (8.6–19.7)	10.4 (5.3–20.9)	0.52
Nevirapine timing data available, *n* (%)	59 (76%)	18 (75%)	0.84
Blood for PCR was collected before NVP, *n* (%)	4 (7)	3 (17)	0.34
Median age (days) at final confirmation of HIV status (IQR)	2 (1–8)	32 (14–180)	<0.0001
Lost to follow‐up, *n* (%)	3 (4)	3 (13)	0.14
Final status, *n* (%)			
Confirmed infected	74 (99)	9 (43)	<0.0001
Confirmed uninfected	0	8 (38)	
Uncertain	1 (1)	4 (19)	

^a^Median time of maternal VL (0.2 weeks after delivery (IQR: 0–2)) and CD4 (5 weeks before delivery (IQR: 15 weeks before–0.2 weeks after)) blood draws relative to delivery were not significantly different between the groups for each test. PCR: polymerase chain reaction; ART: antiretroviral therapy; IQR: inter‐quartile range.

Cases a–d (Figure 1(a)) are all examples of confirmed infected infants where the diagnosis took longer to make on account of confirmatory indeterminate test results that were unable to provide an immediate definitive diagnosis. In all four cases, infants repeatedly tested HIV‐1 PCR indeterminate associated with low‐grade viraemia (around 2–3 log copies/ml).

The HIV‐1 status of cases e–i remains uncertain (Figure 1(b)) because virological detection was noted in at least two separate samples, by virtue of a combination of positive or indeterminate HIV‐1 PCR results and low but quantifiable or indeterminate VL results. However, the virological test results have not fulfilled the criteria for a positive HIV‐1 infection status. The exception is case f who demonstrated virological detection on a single sample at birth that tested HIV‐1 PCR indeterminate with a VL of 2.82 copies/ml on DBS. On account of the uncertain diagnosis associated with a single quantifiable virological result in this case, genetic profiling was performed which confirmed that the birth sample belonged to the correct patient and ruled out the possibility of a sample swap or contamination with another patient's sample. Cases f and g are similar in that both had an HIV‐1 PCR indeterminate result at birth that was associated with a quantifiable VL, using leftover whole blood stored on a DBS card from the birth sample. Both cases were not exposed to maternal ART during the antenatal period. In case g the indeterminate result at birth was followed by an indeterminate result at 10 days of age (during ddNVP exposure) and an indeterminate VL at 35 days of age that was detectable but not quantifiable (<2 log copies/ml). All subsequent virological testing was negative. In both cases f and g, ART was stopped at 76 weeks of age and both have since had at least two undetected virological test results at ±4 weeks off treatment. In case h, a birth indeterminate result was followed 270 days later by a single indeterminate VL and this case is being monitored closely. Case e, who was not followed up on site, had three indeterminate results after the birth PCR positive result, the first of which was tested on a DBS card and occurred during ddNVP exposure while the latter two occurred during combination ART exposure. To date, the patient has never had a quantified VL result on ART. Whereas case i is the only case with an uncertain diagnosis who was not initiated on combination ART, treatment was subsequently stopped for cases f–h on account of inadequate evidence to confirm a positive HIV‐1 infection status. None of these cases had shown any evidence of rebound.

## Discussion

Whereas HIV‐1 status could be confirmed in the majority of neonates who received a non‐negative HIV‐1 PCR result, there were significant delays and challenges associated with infants who tested indeterminate at birth, comprising 24% of all non‐negative screening tests. This group of neonates were found to carry a significant risk of having a positive HIV‐1 infection status, confirmed in 43% of cases, and required extensive follow‐up beyond a standard once off confirmatory test. Furthermore, four neonates who tested indeterminate at birth remain with an uncertain diagnosis. Overall, 25–30% of infants with a non‐negative result required a diagnostic phase of management that extended beyond a single repeat test.

Considering the high morbidity and mortality associated with HIV‐1 infection amongst infants, and considering 4 out of 24 (17%) infants who tested indeterminate at birth died during the course of this study, the need for a rapid definitive diagnosis is clearly of paramount importance. However, with similar proportions of neonates who tested indeterminate at birth found to have a positive and negative HIV‐1 status on subsequent testing, and initiation of treatment prior to confirmatory testing known to confound diagnosis, a balance needs to be struck between effectively managing suspected HIV‐infection and unnecessarily committing a patient to ART. Although NHLS cutoff values used to distinguish positive from indeterminate results are associated with an improved positive predictive value of the assay, they are also necessarily associated with an increase in delayed diagnosis and uncertain HIV‐1 status that requires close monitoring with repeated testing. Furthermore, indeterminate results pose difficulties not only for clinicians but also for primary caregivers and the family of infants given an uncertain HIV‐1 diagnosis. Importantly, these caregivers may lose confidence in clinicians and the healthcare system in general if clinical staff are unable to provide a clear and timely diagnosis.

Although it remains to be determined whether indeterminate HIV‐1 PCR results are more common at birth, indeterminate results are not a phenomenon associated solely with birth testing. Rather they occur within all age groups in which HIV‐1 PCR testing is performed and have been described as a leading cause of non‐negative results within South Africa's EID programme prior to the introduction of birth testing, comprising on average 16% of all non‐negative results [18,32]. More data are needed to assess how birth testing affects the rates of indeterminate results. Whereas concerns surrounding sample swap and contamination are valid, and possibly account for some indeterminate results, they do not comprise the majority of such cases. Similarly, indeterminate results cannot simply be accounted for by citing a reduction in the positive predictive value of diagnostic assays within the context of declining mother‐to‐child transmission rates. Clearly, other factors are associated with uncertain and delayed diagnosis amongst HIV‐infected neonates. Our study demonstrates a correlation between lower maternal and infant VL results in relation to indeterminate HIV‐1 PCR results, suggesting that mechanisms of virological control, including ART and immunological factors, need to be considered when dealing with EID challenges. This further highlights the importance of time of testing, especially considering cases which tested positive at birth but received indeterminate confirmatory results during ART exposure. Sensitivity and specificity of HIV‐1 PCR assays for EID were not measured in our study but sensitivity appears to be decreased by maternal and infant prevention of mother‐to‐child transmission prophylaxis [15–17], and the high proportion of indeterminate results in our study suggests a need for more sensitive and specific assays.

A total of 5 infants (5%), out of 102 neonates with a non‐negative HIV‐1 PCR result at birth, remain with an uncertain diagnosis. Three of these cases (f, g and h) tested indeterminate at birth and were started on ART on the day of confirmatory testing. This practice was based on findings from the previous version of the current assay that neonates with a non‐negative HIV‐1 PCR result at birth, whether positive or indeterminate, were invariably found to have a confirmed HIV‐1 positive infection status [27]. However, once it was determined that this was not the case with the more sensitive CAP/CTM v2.0 assay, this practice was stopped. All three of these infants have since followed up on site, and treatment has been interrupted under close clinical supervision. The diagnosis of these infants remains uncertain as it has yet to be determined what the required length of time is for monitoring post‐treatment cessation in order to exclude HIV‐1 infection [24]. Case e represents the only infant with an uncertain HIV‐1 status where combination ART has not been stopped and is also the only case, amongst those with an uncertain diagnosis, that tested HIV‐1 PCR positive at birth. It is worth noting that this infant is being followed up outside of the study setting and that confirmatory testing was performed on a DBS sample only, without a simultaneous VL test, and that the volume of blood tested on a DBS sample (approximately 60 μl) is less than that used to test EDTA anti‐coagulated whole blood samples (100 μl) and this may have had an impact on the confirmatory result.

As a collective, the diagnostic challenges described in this study raise important questions concerning EID, including the potential for antiretroviral prophylaxis to be associated with virological control and even “functional‐cure”‐type scenarios [9]. Furthermore, infants with multiple indeterminate virological results followed by loss of detectability raise fundamental questions regarding the mechanism of post‐exposure prophylaxis and the possibility of transient or abortive infectious processes. Similarly, it remains to be determined whether isolated indeterminate results represent false detection or true infection associated with faster virological control.

## Conclusions

Whereas the majority of neonates with a positive HIV‐1 PCR test at birth were confirmed to be HIV‐1 infected, indeterminate results were associated with uncertainty and diagnostic delay. Although indeterminate results comprised 24% of all non‐negative birth tests in this study, true ongoing diagnostic dilemmas were rare with most cases resolving on repeat testing, and approximately half of these having a confirmed positive diagnosis and half confirmed negative. In some of these cases a quantifiable VL result confirmed the diagnosis whereas the repeat HIV‐1 PCR test was indeterminate, suggesting a combination of virological testing methods may be beneficial when confirming HIV‐1 infection. Essentially, the clinical requirements and social consequences of managing an infant with an indeterminate HIV‐1 result make it critical that a timely and unequivocal diagnosis is established by the treating clinician and effectively communicated to the primary caregiver.

## Competing interests

The authors have no conflicts of interest to disclose.

## Authors’ contributions

KT, LK, AC, GGS, RS, CTT, EJA: study design; KT, RS, FP, MC, NM, LH, AHM: data collection; KT, AHM, LK, GGS, RS, CTT, PMM, SS, EJA, FP, LH: data interpretation and analysis; KT, AHM, GGS, AC, LK, PMM, SS, LH, RS, FP, MC, NM, CTT, EJA: writing.

## Acknowledgements

We gratefully acknowledge the hard work of Perry Hlalele for work in the laboratory as well as the clinicians at Rahima Moosa Mother and Child hospital for their contribution to the management of patients, particularly Drs Gill Sorour and Gary Reubenson. The study would not have been possible without the willing participants and tireless work of a dedicated study team. The study was supported in part by the National Institutes of Health U01 HD080441, PEPfAR/USAID and UNICEF. We thank the Data Safety and Monitoring Board (DSMB) of the associated LEOPARD study for reviewing some of these cases.

## Funding

This work was supported in part by the National Institutes of Health U01 HD080441; PEPfAR/USAID; and UNICEF.
